# Diffusion-weighted imaging and apparent diffusion coefficient mapping of head and neck lymph node metastasis: a systematic review

**DOI:** 10.37349/etat.2022.00110

**Published:** 2022-12-13

**Authors:** Maria Paola Belfiore, Valerio Nardone, Ida D’Onofrio, Antonio Alessandro Helliot Salvia, Emma D’Ippolito, Luigi Gallo, Valentina Caliendo, Gianluca Gatta, Morena Fasano, Roberta Grassi, Antonio Angrisani, Cesare Guida, Alfonso Reginelli, Salvatore Cappabianca

**Affiliations:** 1 Department of Precision Medicine, University of Campania “L. Vanvitelli”, 80138 Naples, Italy; 2 Unit of Radiation Oncology, Ospedale del Mare, 80138 Naples, Italy; Hospital Clinic Barcelona, Spain

**Keywords:** Diffusion-weighted imaging, magnetic resonance imaging, head and neck cancer, head and neck squamous cell cancer, lymph node

## Abstract

**Aim:**

Head and neck squamous cell cancer (HNSCC) is the ninth most common tumor worldwide. Neck lymph node (LN) status is the major indicator of prognosis in all head and neck cancers, and the early detection of LN involvement is crucial in terms of therapy and prognosis. Diffusion-weighted imaging (DWI) is a non- invasive imaging technique used in magnetic resonance imaging (MRI) to characterize tissues based on the displacement motion of water molecules. This review aims to provide an overview of the current literature concerning quantitative diffusion imaging for LN staging in patients with HNSCC.

**Methods:**

This systematic review performed a literature search on the PubMed database (https://pubmed.ncbi.nlm.nih.gov/) for all relevant, peer-reviewed literature on the subject following the preferred reporting items for systematic reviews and meta-analyses (PRISMA) criteria, using the keywords: DWI, MRI, head and neck, staging, lymph node.

**Results:**

After excluding reviews, meta-analyses, case reports, and bibliometric studies, 18 relevant papers out of the 567 retrieved were selected for analysis.

**Conclusions:**

DWI improves the diagnosis, treatment planning, treatment response evaluation, and overall management of patients affected by HNSCC. More robust data to clarify the role of apparent diffusion coefficient (ADC) and DWI parameters are needed to develop models for prognosis and prediction in HNSCC cancer using MRI.

## Introduction

Head and neck cancer (HNC) is the ninth most prevalent tumor worldwide, and over 90% of all HNCs are squamous cell carcinomas that originate from the epithelium of the mucosal lining [1]. After local therapy as well as in the staging process needed to start a definitive treatment, the development of cervical lymph node (LN) metastases is the key factor affecting patient prognosis [2, 3]. Standard treatment consists of surgery, radiotherapy, chemotherapy, or a combination thereof, depending on the stage, as defined by the American Joint Committee on Cancer Tumor-Node-Metastasis (TNM) staging system [4]. The mainstay in the surgical management of metastatic HNC has conventionally been neck nodal dissection [5]. The presence of LNs with metastatic deposits is associated with poor prognosis, which is about 50% worse than in patients with equivalent tumors but without nodal involvement. Neck LN status is, therefore, the major indicator of prognosis in HNCs, and the early detection of LN involvement, the evidence of necrotic deterioration within infiltrated lymphatic tissue and/or the extra-nodal extension (ENE) have important implications in terms of therapy and prognosis [6]. Accurately staging cervical LNs is essential for planning further clinical management, not least in offsetting any potential curative effect against the risk of morbidity or complications resulting from neck dissection [7–10]. Fine needle aspiration (FNA) biopsy with or without ultrasound (US) guide, may be appropriate for easily accessible nodes to investigate. When US is not exhaustively showing a clear LN involvement, computer tomography (CT) scan and magnetic resonance imaging (MRI) are useful imaging modalities in the initial assessment and diagnosis of HNC, and both have been routinely used to guide the choice of therapy as well as in patient monitoring and follow-up after treatment [11–13].

While the assessment of the primary site can be performed with CT of the soft tissues of the neck or MRI of the neck, MRI is preferred in specific conditions such as oral cavity cancer, nasopharyngeal cancer, sinonasal cancer, and to complete the LNs evaluation. A positron emission tomography (PET)/CT scan may be required for distant metastasis detection, in the staging process.

Key factors correlating with LN involvement include morphological features such as size, shape, internal biological components (e.g., necrotic aspects), vascularity, and ENE. However, the accuracy of the routinely used imaging tools is somewhat limited, and some limitations still persist. CT scan is routinely used for diffusion-weighted imaging (DWI) and is a non-invasive imaging technique able to characterize tissues based on the displacement motion of water molecules (Brownian motion) [14–17]; the range of motion is distinguished by its apparent diffusion coefficient (ADC) values [18, 19]. Loss of signal in diffusion sequences is caused by the motion of water molecules, which leads to phase dispersion of the spin; the ADC map can measure the amount of signal loss within the biological tissue [20, 21]. Several studies used DWI to evaluate cervical LNs in head and neck imaging [22–25]. This review aims to provide an overview of the current literature concerning quantitative diffusion imaging for nodal staging in patients with head and neck squamous cell cancer (HNSCC).

## Materials and methods

This systematic review was conducted following the preferred reporting items for systematic reviews and meta-analyses (PRISMA) statements [26]. First, it was performed a literature search to identify studies published in English and registered on Embase, PubMed, and ScienceDirect. We used January 2009 as the start date, and the search result was last updated in January 2022. We used a search string containing free text and/or Medical Subject Headings (MeSH) with the following main search terms: “DWI”, “MRI”, “head and neck”, “staging”, and “lymph node”. Duplicate results were weeded out and all references cited in the retrieved results were evaluated.

Eligibility criteria. The inclusion criteria were: a) English articles, and b) original articles. Exclusion criteria were: a) case reports, b) review articles, c) poster presentations, and conference abstracts.

All articles were independently identified by two reviewers (Maria Paola Belfiore, and Luigi Gallo) with an experience of at least 5 years in HNSCC, based on the above criteria. Possible disagreements were resolved after a panel discussion with two other authors familiar with the project (Valerio Nardone and Alfonso Reginelli).

Quality assessment and data analysis. Two reviewers (Valerio Nardone and Antonio Angrisani) with proven expertise in systematic reviews with or without meta-analysis independently assessed the quality of the studies with the quality assessment of diagnostic accuracy studies-2 (QUADAS-2) tool [27]. Disagreements between reviewers were resolved by consensus.

QUADAS-2 tool was used to quantify the accuracy of data extracted from the eligible studies. According to the four main domains (patient selection, index test, reference standard, flow, and timing of patients’ diagnostic path), a score for each domain was used to analyze results from each group of studies. Although a standardized metric was not used, such a tool helped reviewers to judge the eventual bias and applicability of the studies. A critical appraisal of the relevant data collection was carried out.

## Results

**Figure 1.**
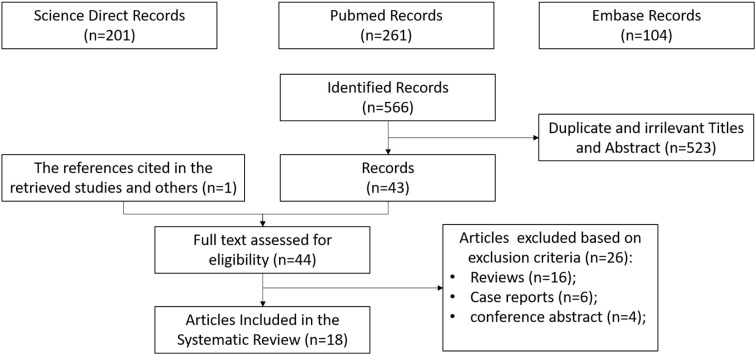
PRISMA flow chart of the eligible studies after identification and screening of citations

A total of 567 records were identified by the date of the search (January 15, 2022). Duplicates were removed and, after screening titles and abstracts not related to the topic of interest, 523 citations were excluded. One reference was added after a hand-search, while 26 screened articles were excluded because they were not original. Eighteen studies fulfilled the eligibility criteria and were included in this systematic review. A flow chart of the search process is shown in Figure 1.

The synthesis of collected data involved a total of 675 patients, however, after careful and critical analysis, the studies’ heterogeneity did not enable a direct, consistent, and robust synthesis of such data. Nine out of eighteen studies were prospective, eight reports were performed post-treatment, while twelve evaluated the HNSCC stage before any kind of therapy. Six studies were designed to conclude DWI values evaluation. Given such heterogeneity, the MRI modalities used, and the relative results were synthesized in form of tables.

**Figure 2.**
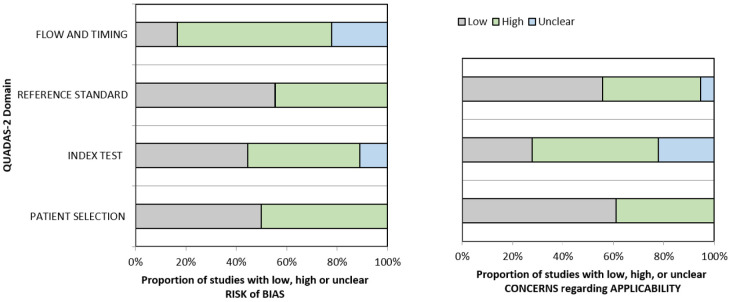
Grouped bar graphs of risk of bias and applicability concerns for the included diagnosis-related studies using QUADAS-2

Quality analysis of the included studies. A graphical display of QUADAS-2 results for the 18 included studies is shown in Figure 2. In terms of patient selection, 9 studies (50%) were found to be at high or unclear risk of bias due to the ambiguity of the inclusion criteria. Regarding the index test, 10 studies (55.5%) were classified as high or unclear because no threshold was chosen and there was no blinding between the reference standard and the index risk itself. The risk of bias due to the reference standard and flow and timing was classified as high or unclear in 8 studies (44.4%) and 15 studies (83.3%), respectively, because of uncertainties in the reference standard. The applicability of QUADAS-2 was also unclear or highly questionable in the domains of the reference standard (7 studies, 38.8%), index test (13 studies, 72.2%), and patient selection (8 studies, 44.4%).

MRI characteristics of the included studies. Among the 18 relevant papers, the b-values used in the studies we reviewed were b0–1000 [12, 16, 28–31], b500–1000 [32–35], b0–800 [36–40], b250–1000 [41], and b0–1500 [42, 43]. In Table 1 protocols, machines, and standard references used are shown.

**Table 1.** Summary of the included studies, protocol/machine used, and standard reference

**Study**	**b-value**	**MRI protocol and machine**	**Reference standard**
Vandecaveye et al. [28]	b0–1000	1.5Tesla (T) MRI TSE (T1,T2) + DW sequences	Histopathology
Dirix et al. [29]	b0–1000	MRI (T1,T2 + DW sequences)	Histopathology
Perrone et al. [32]	b500–1000	1.5T; FSE T1, T2 sequences	Histopathology
Nakamatsu et al. [30]	b0–1000	1.5T MRI SSEP; DW sequences	Histopathology
Taha Ali [16]	b0–1000	1.5T MRI T1, T2-weighted sequences	Histopathology
Lee et al. [12]	b0–1000	3T MRI TSE (T1,T2) + DWI sequences	Histopathology
Hauser et al. [36]	b0–800	3T MRI, DW sequences	Mixed or unclear
Heusch et al. [33]	b500–1000	3T MRI, STIR TSE; T1-weighted TSE sequence + DWI sequences	Histopathology
Hoang et al. [41]	b250–1000	1.5T MRI STIR T1-weighted + DWI sequences	Clinical
Chen et al. [37]	b0–800	3T MRI DWI STIR technique	Clinical
Schouten et al. [34]	b500–1000	1.5T MRI STIR T1-weighted + DWI sequences	Histopathology
Stecco et al. [38]	b0–800	1.5T MRI STIR T1-weighted + DWI sequences	Mixed clinical and histop
Jin et al. [39]	b0–800	1.5T MRI TSE(T1,T2) + DWI sequences	Histopathology
Park et al. [31]	b0–1000	3T MRI TSE(T1,T2) + DWI sequences	Clinical
Freihat et al. [40]	b0–800	3T MRI TSE(T1,T2) + DWI sequences	Histopathology
Abdel Razek et al. [24]	b500–1000	1.5T MRI T1, T2 + DWI sequences	Histopathology
Anjari et al. [42]	b0–1500	1.5T MRI T1, T2 + DWI sequences	Mixed or unclear
Connor et al. [43]	b0–1500	1.5T MRI STIR T1, T2 + DWI sequences	Histopathology

TSE: turbo spin echo; FSE: fast spin echo; STIR: short tau inversion recovery; SSEP: somatosensory evoked potentials; DW: diffusion weighted

Vandecaveye et al. [28] performed a prospective study on 33 patients diagnosed with HNSCC who were subjected to TSE MRI and DW-MRI examination, with blinding to the DW images; Taha Ali [16] performed a prospective study on 34 patients with suspicious neck LNs who underwent an MRI (including DW sequences) examination before histopathological analysis of the nodes. The patients were divided into three subgroups based on the pathological examination: benign, metastasis, and lymphoma. Both these studies came to similar conclusions on the relevance of ADC values to revealing malignant LNs, although patient selection and stratification were different. Conversely, Nakamatsu et al. [30] performed a retrospective study on 24 patients affected by HNSCC who had undergone both MRI and PET/CT examinations between 2007 and 2010 before any treatment and reported opposite results.

Dirix et al. [29] performed a prospective study on 22 patients who underwent both a CT and an MRI (including DW sequences) scan for previously untreated HNSCC. Regions of interest (ROIs) were placed on DW images by two experienced radiologists, in reciprocal consensus, around identifiable LNs. Perrone et al. [32] performed a study on 32 patients undergoing MRI exams including DW sequences. Whole node ADC values were calculated by drawing ROIs and calculating the average value; Lee et al. [12] performed a study on 22 patients scheduled to undergo surgical treatment of biopsy-proven HNSCC; the patients underwent an MRI examination including standard TSE and DW sequences before undergoing surgery. Hauser et al. [36] performed a study on 15 patients affected by HNSCC to obtain diffusion and microperfusion measures in LN metastases of HNSCC; the patients underwent an MRI examination before receiving chemoradiotherapy (CRT). Heusch et al. [33] performed a study on 18 patients—14 with suspected HNSCC and 4 with known HNSCC and suspected nodal relapse—who were examined before undergoing surgical treatment; these patients underwent both an 18 fluorodeoxyglucose (FDG)-PET/CT and an MRI examination (including DW sequences). Hoang et al. [41] performed a study to evaluate inter-patient variability in ADC values for HNSCC and compare it with early treatment-induced changes in these values. Sixteen patients were prospectively enrolled and underwent two baseline MRI scans (including DW sequences) at a 1-week interval followed by a third after the second week of CRT. Chen et al. [37] performed a study involving 35 patients with newly diagnosed nasopharyngeal carcinoma (NPC), having received no prior treatment and being referred for CRT. Schouten et al. [34] performed a retrospective study on a cohort of 84 patients histologically diagnosed with advanced stage HNSCC; after 3 months of CRT, these patients were subjected to DW-MRI and 18FDG-PET/CT examinations. Stecco et al. [38] performed a retrospective study on a cohort of 25 patients with HNSCC who had undergone MRI and PET/CT scans before treatment; the authors assessed the sensitivity, specificity, positive and negative predictive values of PET/CT, MRI, and their combined use in T and N staging. Jin et al. [39] performed a study on a cohort of patients affected by NPC to evaluate the efficacy of DW-MRI in assessing the benign or malignant nature of cervical LNs smaller than 10 mm: all of the patients underwent a DW-MRI scan using a 1.5T device; after no more than 2 weeks an US-guided FNA biopsy (FNAB) was performed on selected LNs. Park et al. [31] performed a retrospective study evaluating the diagnostic performance of texture analysis using ADC data of multi-shot echoplanar imaging-based DWI in predicting metastatic LNs of HNSCC. Their study included 36 patients, all of whom had undergone a pre-treatment MRI examination at 3T, including multi-shot echo-planar-DWI (msEPI-DWI) and 18FDG- PET/CT. Freihat et al. [40] performed a retrospective study of 90 patients, all of whom had undergone an 18FDG-PET scan and a DW-MRI scan (b-values of 0 and 800 mm2/s). Sixty-five of these patients had been diagnosed with HNSCC with LN metastases; the other 25 patients were randomly selected healthy patients from their radiology department database; a cervical LN was selected in the scans of the 25 healthy patients for the study. The ADC map generated from the DWI scans was used to measure ADC values; nodal ADC values were obtained by drawing an ROI covering the greatest possible area of the most solid and/or homogenous part. Razek and Helmy [35] performed a retrospective study on 43 patients with primary or recurrent HNSCC who later underwent neck dissection; LN status was then finally confirmed by histopathological examination. Before undergoing neck dissection, the patients were subjected to an MRI exam including arterial spin labeling and DW sequences. Image examination was performed by two radiologists who specialized in head and neck imaging. Anjari et al. [42] performed a prospective cohort study of 25 stages 3–4 HNSCC patients who underwent a DW-MRI examination pre-treatment and again at 6- and 12-week post-CRT. Connor et al. [43] analyzed a cohort of 56 patients affected by HNSCC; they performed a preliminary DW-MRI scan of these patients before CRT; 12 weeks after the end of CRT they then performed both a DW-MRI (ADCmax and ADCmin) and an 18FDG-PET [standardized uptake value (SUV); SUVmax and SUVmin ratio to the liver] examination and compared the results.

In Table 2 the main results of the included studies are summarized along with the mean follow-up time, when available or applicable.

**Table 2.** Insight into the main retrieved findings

**Study (study type)**	**Follow-up time (sample size)**	**Main results**	**Direct conclusions**
Pre-treatment LNs evaluation			
Vandecaveye et al. [28] (Prospective)	Not applicable (33 patients)	ADC values are significantly lower for metastatic LNs than for benign ones	LN > 1 cm: ADC > TSE evaluation to select benign LNs LN < 1 cm: ADC showed higher sensitivity but slightly lower specificity
Perrone et al. [32] (Retrospective)^*^	Not applicable (32 patients)	A statistically significant relationship exists between DWI and ADC findings, as well as the nodal status	DW acquisitions would be an asset in both identifying pre-treatment nodal status and in evaluating treatment response. Not stratified patients/histology
Nakamatsu et al. [30] (Retrospective)	Not applicable (24 patients)	A statistically significant relationship exists between DWI and ADC findings, as well as the nodal status	DW acquisitions would be an asset in both identifying pre-treatment nodal status and in evaluating treatment response. Not stratified patients/histology
Taha Ali [16] (Prospective)	Not applicable (34 patients)	Correlation between ADC values and LN status	ADC values alone offer no statistically significant information concerning the grade of LN metastasis
Hauser et al. [36] (Retrospective)	13.5 months (15 patients)	Benign LNs had significant ADC values compared to malignant ones, and the ADC values of metastatic LNs were significantly higher than those of LNs affected by lymphoma	The ADC values of LNs affected by well-differentiated metastasis were then significantly higher than those of LNs with poorly differentiating metastasis. (similar conclusion of vandecaveve, but with a different sample)
Heusch et al. [33] (Retrospective)^*^	Not applicable (18 patients)	No significant difference in diagnostic accuracy concerning nodal status between 18FDG-PET/CT and 18FDG-PET-MRI, nor between 18FDG-PET/CT and 18FDG-PET-MRI + DWI	US has greater diagnostic accuracy compared to 18FDG-PET/CT, but not compared to 18FDG-PET/CT and 18FDG-PET-MRI + DWI. The addition of DWI to the diagnostic process significantly improves the detection of metastases in normal-sized LN
Stecco et al. [38] (Retrospective)^*^	15 months (25 patients)	Using both DW-MRI and PET/CT increases the diagnostic value of T and N parameters	DW-MRI and PET/CT must be combined for the HNSCC work-up
Jin et al. [39] (Prospective)	Not applicable (65 patients)	A statistical correlation does exist between mean ADC values of cervical LN and their benign or metastatic nature	
Park et al. [31] (Retrospective)	Not applicable (36 patients)	Several features from the first- and second-order whole lesion volumetric texture analysis of ADC data using msEPI-DWI were significantly different between metastatic and benign LNs in HNSCC	TA values providing esteem for complexity, energy, and roundness were significant predictive factors for nodal metastases. Complexity was the single best predictive feature
Freihat et al. [40] (Retrospective)	Not applicable (90 patients)	A statistically significant difference does exist between metastatic and normal LNs’ ADC values	
Pre-/post-treatment evaluation			
Dirix et al. [29] (Prospective)^*^	Not applicable (22 patients)	The superiority of DWI imaging compared to conventional imaging in nodal staging	DWI imaging best allows us to correctly assess both GTV and CTV of nodal lesions, which in turn allows for a more precisely targeted RT, sparing healthy tissues
Lee et al. [12] (Prospective)	Not applicable (22 patients)	Low impact of ADC value cutoff for differentiating benign and malignant LNs	DWI examination provided greater sensitivity and specificity than conventional TSE MRI
Hoang et al. [41] (Prospective)	31 months (16 patients)	Intra-treatment variability in ADC values for metastatic LNs is greater than their baseline variability	Inherent baseline variability should be kept into account to ensure a more accurate evaluation of treatment-induced changes in ADC
Chen et al. [37] (Prospective)	< 6 months (35 patients)	ADC increases were significantly larger in responders than in non-responders; however, in patients with NPC, the pre-treatment ADCs of responders to NAC were not significantly lower than those of non-responders	
Schouten et al. [34] (Retrospective)^*^	Not applicable (84 patients)	PET/CT recognized all regional residues and DW-MRI recognized most patients with regional control with substantial and moderate observer agreement, respectively	
Razek et al. [35] (Retrospective)	Not applicable (43 patients)	Combining TBF and ADC evaluation may yield the best results in evaluating LNs	
Anjari et al. [42] (Prospective)	< 6 months (25 patients)	Metastatic LNs have higher TBF and lower ADC values, compared to reactive ones and such differentiation is easier when both techniques are combined	TBF and ADC evaluation may yield the best results in evaluating LN status
Connor et al. [43] (Prospective)^*^	Not available (56 patients)	No statistically significant or clinically relevant correlation between DW-MRI and 18FDG-PET scan results 12 weeks after CRT for LNs. Clinically relevant DWI changes for primary tumors response evaluation	Therapy efficacy assessment ADC mean at 12 weeks post-CRT DW-MRI (*P* = 0.03 ) and the interval change in nodal ADC min from pre-treatment to 12 weeks post-CRT DW-MRI (*P* = 0.05 ) were associated with 2-year DFS

* Reports focused on DWI evaluation. TBF: tumor blood flow; GTV: gross target volume; CTV: clinical target volume; RT: radiotherapy; DFS: disease free survival

## Discussion

The basic concept underlining DWI is to generate images by assessing differences in the motion of water molecules in different tissues; this technique provides quantitative and qualitative information about tissues, which is useful in detecting their nature [44]. The role of DWI in the management of malignancies has been increasingly relevant in recent years [45, 46]. DWI was first used in neuroimaging [47], but its role has since expanded to encompass the diagnostic management of a wide range of pathological conditions [48–50]. The heterogeneity in study designs, the acquisition protocols used, as well as the timing of the imaging performed was surprisingly high among the selected studies for the current analysis, thereby solid conclusions are lacking. However, most of the literature examined here suggested that using DWI when assessing the status of LNs in patients affected by HNSCC is a sound and effective practice. The results of the reviewed papers mostly show that DWI improves the diagnosis, treatment planning, treatment response evaluation, and overall management of patients affected by HNSCC and that DWI findings and related ADC map patterns are significantly different in normal *vs.* metastatic LNs (Table 1). Despite that, several drawbacks of this method must be resolved before applying it in clinical practice.

The magnitude of bias introduced with the reference standard assessment, which has been found quite inhomogeneous among the evaluated studies, has its own impact on the final interpretation of these results. In 11 studies histopathologic assessment of LNs was used as a standard evaluation of suspicious LN. Differently, a clinical approach (imaging only) was chosen in 3 studies, and a mixed approach (arbitrary choice of histopathology or clinical assessment) or an unclear standard reference was found in 4 eligible citations. In light of the QUADAS-2 assessment regarding the applicability of such results, given the importance of a shared and robust reference standard, it would be advisable for future studies as well as future reviews on this topic, to avoid unclear information on the reference standards. Moreover, a wide explanation of the rationale underneath the choice of the reference standard could be beneficial for the final interpretation. Also, all the studies included in the meta-analysis do not foresee the external validation of the results obtained in terms of the prediction of N status in an external dataset.

The QUADAS-2 assessment resulted particularly low in terms of patient selection due to ambiguity of the inclusion criteria in nine studies (50%), whereas in 10 studies (55.5%) no threshold was chosen. The risk of bias due to flow and timing was also extremely high in 15 studies (83.3%). All these concerns limit the applicability of this method in several domains of the QUADAS-2 scale.

The currently available literature also suggests that DWI is an effective asset for the follow-up of patients with HNSCC, as it provides a satisfactory differentiation between reactive and metastatic LNs [35, 42]. DWI also showed high positive and negative predictive values for nodal status [39]. Some studies compared the diagnostic accuracy of DWI with that of 18FDG-PET/CT [30, 31, 33, 34, 38, 40, 43], or the diagnostic advantage obtained by combining the two, which is also common practice in the evaluation of other malignancies [51–54]. The overall results reveal a degree of ambiguity in this regard, and further studies will be required to clarify the issue, although one study did find that combining the results from DWI and 18FDG-PET/CT increases both positive and negative predictive values for T and N parameters [38]. One study suggested that textural complexity might be the single most important DWI feature for determining nodal status [31]. Layering DWI features to assess which might be the most important for nodal staging is an interesting hypothesis that has not yet been widely explored, at least among the papers we reviewed. To date, no studies have supported the idea of using DWI as the only tool for the evaluation of head and neck nodal status, and one article reports that ADC values alone offer no information concerning the grade of LN metastases [30]. In opposition to the majority of the mentioned results, only one study found no significant difference in the ADC values of metastatic LNs of patients affected by HNSCC with either good or bad prognoses [36].

Our systematic literature review was done following PRISMA items, with rather wide inclusion criteria, allowing for a relatively high number of citations selected. A limitation for the review could be found in the exclusion criteria, which could be stricter as well as more direct to answer the scientific question, therefore allowing for a smaller number of reports eligible for synthesis.

Further investigations may be useful in exploring whether some DWI features of metastatic LNs could provide valuable prognostic information. Currently, other tools are available for evaluating nodal status in patients with head and neck malignancies, mainly traditional MRI sequences and 18FDG-PET/CT. However, compared to conventional MRI sequences, DWI offers a quantifiable set of data, with the possibility of establishing ADC value thresholds to assess the benign or malignant nature of nodal lesions [31]; compared to 18FDG-PET/CT, DW-MRI has the crucial advantage of not involving the management and administration of radioactive compounds, with all the associated costs and risks for both patients and healthcare professionals. DWI is also usually performed along with traditional MRI sequences offering higher resolution; more advanced MRI devices operating with higher intensity magnetic fields offer the prospect of higher resolution images [55]. Finally, the use of artificial intelligence (AI)-based solutions in healthcare has already begun to change established paradigms across the entire healthcare sector [56–60]. Further studies will also be needed to address the relatively new field of radionics, in particular as applied to MRI and DWI.

## Abbreviations

ADC: apparent diffusion coefficient
CRT: chemoradiotherapy
CT: computer tomography
DWI: diffusion-weighted imaging
HNC: head and neck cancer
HNSCC: head and neck squamous cell cancer
LN: lymph node
MRI: magnetic resonance imaging
NPC: nasopharyngeal carcinoma
PET: positron emission tomography
PRISMA: preferred reporting items for systematic reviews and meta-analyses
QUADAS-2: quality assessment of diagnostic accuracy studies-2
ROIs: regions of interest
TSE: turbo spin echo
US: ultrasound

## Declarations

### Copyright

© The Author(s) 2022.
